# Ruxolitinib for rheumatic disease-associated macrophage activation syndrome in children: a retrospective cohort study

**DOI:** 10.3389/fimmu.2026.1852538

**Published:** 2026-08-18

**Authors:** Xiaona Zhu, Mengmeng Zhang, Jun Yang, Tingyan He

**Affiliations:** Department of Rheumatology and Immunology, Shenzhen Children’s Hospital Affiliated Shenzhen University, Shenzhen, China

**Keywords:** JAK inhibitor, macrophage activation syndrome, rheumatic disease, ruxolitinib, treatment

## Abstract

**Objective:**

Macrophage activation syndrome (MAS) is a severe, life-threatening complication in pediatric rheumatic diseases. Current treatments are predominantly based on glucocorticoids; however, their long-term use is associated with significant adverse effects. Ruxolitinib, a Janus kinase (JAK) inhibitor, has emerged as a potential treatment option, but clinical evidence in pediatric populations remains limited. This study aimed to evaluate its efficacy and safety in pediatric patients with rheumatic disease-associated MAS (RD-MAS).

**Methods:**

We retrospectively reviewed the medical records of pediatric patients with RD-MAS treated at Shenzhen Children’s Hospital between January 2021 and December 2025. Eligible patients had a confirmed rheumatic disease, fulfilled the 2016 EULAR/ACR classification criteria for MAS, and had complete clinical, laboratory, treatment, and follow-up data. Patients who received ruxolitinib in addition to conventional immunosuppressive therapy were compared with contemporaneous patients treated with conventional immunosuppressive therapy alone. The primary outcome was time to complete remission (CR). Secondary outcomes included remission rates during follow-up, changes in laboratory parameters, glucocorticoid-sparing effect, cumulative glucocorticoid exposure, and adverse events.

**Results:**

A total of 21 patients with RD-MAS were included, of whom 10 received ruxolitinib (ruxolitinib group) and 11 received conventional therapy alone (non-ruxolitinib group). In the ruxolitinib group, 10 patients (6 males, 4 females) had underlying diagnoses of systemic juvenile idiopathic arthritis (sJIA, n = 7), systemic lupus erythematosus (SLE, n = 1), or other connective tissue diseases (n = 2). All patients presented with active disease at baseline. 30% (3/10) of patients achieved CR by week 4, and all patients achieved CR by week 48. By week 8, the median prednisone-equivalent dose decreased from 4 mg/kg/day to 0.4 mg/kg/day, and all patients successfully discontinued glucocorticoids by week 48 while maintaining remission. No significant difference in time to CR or infection rates was observed between groups. However, the ruxolitinib group demonstrated significantly lower cumulative glucocorticoid exposure during follow-up.

**Conclusions:**

Ruxolitinib-based therapy was associated with sustained disease control and a significant glucocorticoid-sparing effect in pediatric RD-MAS. These findings support that ruxolitinib may serve as a promising steroid-sparing strategy with favorable efficacy and safety for pediatric RD-MAS, although larger prospective studies are warranted.

## Introduction

Macrophage activation syndrome (MAS) is a state of immune hyperactivation that can result in life-threatening multisystem organ dysfunction (1). MAS in children most commonly occurs in patients with rheumatic diseases, particularly systemic juvenile idiopathic arthritis (sJIA) and systemic lupus erythematosus (SLE). Key features of MAS are overactivation of T lymphocytes causing highly elevated production of interferon-γ (IFN-γ), which drives activation of hemophagocytic macrophages (2).

Although treatment recommendations are available for MAS complicating sJIA, evidence-based guidelines covering the broader spectrum of pediatric RD-MAS remain limited. Etoposide-based regimens, such as Hemophagocytic Lymphohistiocytosis (HLH)-1994 and HLH-2004 protocols, are widely recognized treatments for primary HLH and severe secondary HLH; however, their use in RD-MAS in RD-MAS is limited due to high toxicity and differences in underlying pathophysiology (3). Glucocorticoids remain the cornerstone of therapy due to their broad immunosuppressive effects, but prolonged high-dose use is associated with significant adverse outcomes. Biologic agents targeting specific cytokines, including anakinra, emapalumab and tocilizumab, have demonstrated potential therapy in case reports and small-scale clinical studies, highlighting the importance of cytokine-directed therapies in MAS (4, 5).

Ruxolitinib, a Janus kinase (JAK) 1 and 2 inhibitor, has emerged as a promising therapeutic option for MAS. By inhibiting the JAK1/2-STAT1 pathway, ruxolitinib can suppress IFN-γ signaling and other key proinflammatory cytokines involved in the pathogenesis of MAS (6). Recent studies have shown that ruxolitinib can effectively ameliorate both clinical and laboratory manifestations in primary HLH and MAS (7). However, the efficacy and safety of ruxolitinib in pediatric patients with RD-MAS remains limited, particularly in real-world clinical settings.

In this study, we conducted a retrospective cohort of pediatric patients with confirmed RD-MAS, including those treated with ruxolitinib and those receiving conventional therapy alone. We evaluated the clinical outcomes, safety, and glucocorticoid-sparing effects, aiming to provide clinically relevant evidence to guide therapeutic decision-making and future treatment strategies.

## Methods

### Patient cohort and study approval

We retrospectively reviewed the medical records of pediatric patients with RD-MAS treated at Shenzhen Children’s Hospital between January 2021 and December 2025. Patients were eligible for inclusion if they met all of the following criteria: (1) a confirmed diagnosis of rheumatic disease; (2) a diagnosis of MAS based on the 2016 EULAR/ACR classification criteria for MAS in sJIA (8); (3) complete clinical and laboratory documentation; (4) detailed treatment information, including drug types, dosages, and administration regimens; (5) a minimum follow-up duration of at least 1 year. Patients with malignancies, monogenic autoinflammatory syndromes, or active infection were excluded. Patients with monogenic autoinflammatory syndromes were excluded because of their distinct pathogenesis and treatment responses. Patients with active infection were excluded to minimize confounding from infection-driven hyperinflammation.

Given the rarity and severity of MAS, randomized controlled trials are difficult to conduct. Therefore, we included patients treated with ruxolitinib in addition to conventional immunosuppressive therapy, with contemporaneous patients receiving conventional immunosuppressive therapy alone (including mycophenolate mofetil, cyclosporine, or methotrexate) serving as the control group. In this retrospective study, the introduction of ruxolitinib was at the discretion of the treating physicians for patients with persistent hyperinflammation, inadequate response to glucocorticoids and/or conventional immunosuppressive therapies, or recurrent disease activity during glucocorticoid tapering. Furthermore, drug accessibility and patients’ financial capacity were key factors influencing the actual use of ruxolitinib.

The study was approved by the ethics committee of Shenzhen Children’s Hospital. A waiver of re-obtaining written informed consent was granted as the research involved minimal risk and used anonymized data.

### Data collection

Clinical data were retrospectively collected from inpatient medical records, including clinical manifestations, laboratory findings, and levels of serum cytokines. Laboratory parameters included white blood cell (WBC) count, neutrophil count, platelet count, erythrocyte sedimentation rate (ESR), hemoglobin, C-reactive protein (CRP), ferritin (FER), and fibrinogen. Serum cytokines included IFN-γ, interleukin (IL)-10, IL-6, tumor necrosis factor-α (TNF-α), IL-4, and IL-2. Treatment regimens included glucocorticoids, immunosuppressive agents, and intravenous immunoglobulin (IVIG). Baseline was defined as the time of RD-MAS diagnosis, prior to initiation of the treatment regimen evaluated in this study, as well as follow-up data at weeks 2, 4, 8, 12, 24, 48, were collected and analyzed.

### Treatment response and safety

Treatment response was classified into three categories: complete remission (CR), partial remission (PR), and no response (NR). CR was defined as resolution of all clinical symptoms and normalization of all parameters. PR was defined as improvement in at least two predefined clinical or laboratory parameters, including a ≥50% reduction in serum ferritin and soluble CD25, as well as at least a onefold increase in neutrophil count, with no progression of other HLH-associated markers. This definition was adapted from previously published response criteria for pediatric hemophagocytic lymphohistiocytosis (HLH) (7). Compared with HLH-2004 response criteria, our definition places greater emphasis on ferritin and neutrophil recovery, which better reflects RD-MAS inflammatory dynamics. NR was defined as failure to meet the criteria for either CR or PR (9). The primary outcome was time to complete remission (CR). Secondary outcomes included laboratory response, glucocorticoid-sparing effect, cumulative glucocorticoid exposure, and adverse events.

### Statistical analysis

Statistical analysis was performed using GraphPad Prism software (version 8.0.1, GraphPad Software, USA). Given the limited sample size, multivariable adjustment was not performed because inclusion of multiple covariates would have resulted in overfitting and unstable estimates. Continuous variables were expressed as median with interquartile range (IQR) and categorical variables were presented as percentages. Comparisons between groups were performed using the Welch’s t-test or Mann-Whitney U test. Categorical variables were compared using the chi-square test or Fisher’s exact test. Time to CR was analyzed using the Kaplan–Meier method, and differences between groups were assessed using the log-rank test. The proportion of patients achieving remission at each time point was calculated, and 95% confidence intervals (CIs) were estimated using the Wilson method. To evaluate longitudinal changes in glucocorticoid dosage, a linear mixed-effects model was applied. Fixed effects included treatment group, time, and the interaction between group and time, while patient ID was included as a random effect to account for repeated measurements. A value of p <0.05 was considered statistically significant.

## Results

### Demographics and clinical characteristics

**Figure 1:**
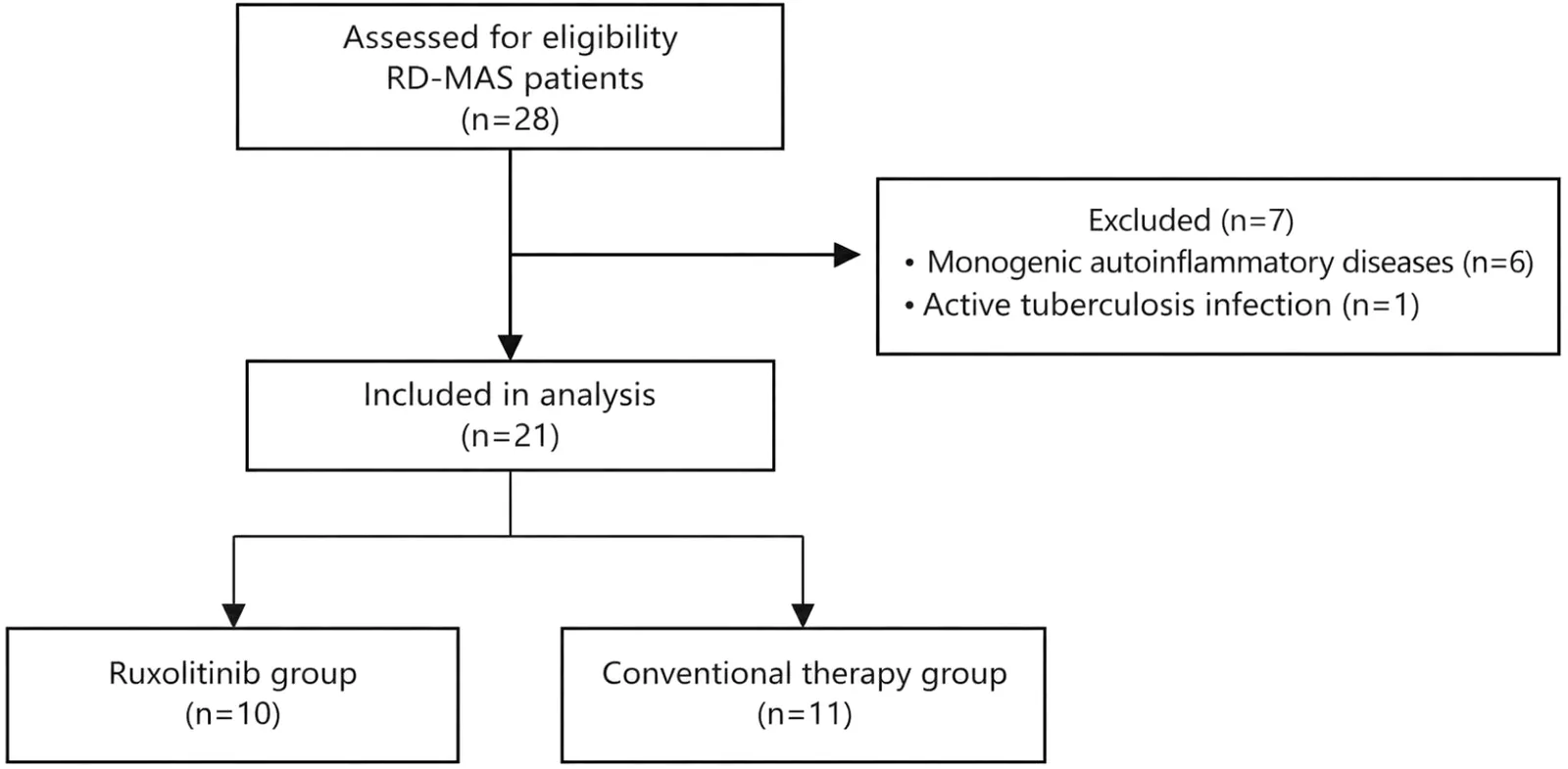
Flowchart of patient selection. RD-MAS, rheumatic disease-associated macrophage activation syndrome.

A total of 21 pediatric patients with RD-MAS were included (ruxolitinib group, n = 10; non-ruxolitinib group, n = 11) (**Figure 1**). The baseline demographic and clinical characteristics are summarized in **Tables 1**, **2**.

**Table 1:** Baseline demographics and clinical characteristics of patients.

Characteristic	Ruxo group (n=10)	Control group (n=11)	P value
Age at disease onset, median (IQR), years	8.9 (3.2, 9.9)	9.2 (4.8, 11.6)	0.661
Male, n (%)	6 (60.0)	3 (27.3)	0.130
Rash, n (%)	9 (90.0)	11 (100.0)	0.283
Lymphadenopathy, n (%)	8 (80.0)	10 (90.9)	0.476
Hepatomegaly, n (%)	4 (40.0)	4 (36.4)	0.864
Splenomegaly, n (%)	1 (10.0)	1 (9.1)	0.943
Arthritis/arthralgia, n (%)	5 (50.0)	3 (27.3)	0.284
Serositis, n (%)	0 (0.0)	3 (27.3)	0.074
White blood cells (109/L) (4.0–10.0)	15.2 (5.5, 21.6)	2.7 (1.8, 13.9)	**0.041**
Neutrophils (109/L) (1.8–7.5)	13.2 (4.2, 19.5)	1.4 (0.8, 13.0)	**0.041**
Hemoglobin (g/L) (110–170)	108 (88, 122.8)	102 (94.0, 114.0)	0.757
Platelets (109/L) (150–388)	222 (167, 469.5)	160.0 (83.0, 277.0)	0.159
CRP (mg/L) (0-10)	99.3 (26.9, 162.9)	7.2 (0.4, 59.3)	**0.029**
ESR (mm/h) (0-15)	39 (17.5, 81.8)	33.0 (10.0, 48.0)	0.196
Ferritin (ng/mL) (14-79)	4112.9 (1451.5, 9741.5)	2158.6 (936.0, 7937.0)	0.324
Triglycerides (mmol/L) (0-1.7)	1.1 (0.8, 1.3)	1.6 (1.2 2.6)	0.053
Fibrinogen (g/L) (1.31-4.51)	4.7 (3.3, 7.8)	2.4 (1.7, 4.2)	**0.021**
ALT (IU/L) (7-30)	36 (13.8, 66.3)	76.0 (34.0, 347.0)	0.078
AST (IU/L) (14-44)	45 (25.0, 124.3)	154.0 (57.0, 825.0)	0.011
Lactate dehydrogenase (IU/L) (170-283)	409.0 (332.8, 523.5)	543.0 (434.0, 814.0)	0.105
sCD25 (U/mL) (223-710)	2900 (918.5, 6274.8)	3315.0 (1185.0, 11538.5)	0.884
IFN-γ (pg/mL) (0-4.43)	28.2 (2.5, 469.9)	2.0 (1.3, 53.5)	0.849
IL-6 (pg/mL) (0-11.09)	29.7 (6.6, 80.8)	11.9 (3.0, 15.1)	0.095
IL-10 (pg/mL) (0-5.61)	5.3 (2.4, 15.3)	10.3 (2.9, 19.6)	0.342
Hemocytopenia of two or three lines, n (%)	3 (30.0)	5 (45.5)	0.466
Hemophagocytosis images on bone marrow aspirates, n (%)	2 (20.0)	4 (36.4)	0.407
Meet the HLH-2004 diagnostic criteria, n (%)	1 (10.0)	4 (36.4)	0.157
Etiology			
SLE, n (%)	1 (10.0)	5 (45.5)	
sJIA, n (%)	7 (70.0)	3 (27.3)	
Other CTDs, n (%)	2 (20.0)	3 (27.3)	
Initial treatment of GCs (equivalent dose of Pred, mg/kg/day)	4 (2, 12.5)	2 (2, 3)	0.062

Values in parentheses in the first column indicate the reference range for each laboratory parameter. Continuous variables were expressed as median with interquartile range (IQR) and categorical variables were presented as percentages. CRP, C-reactive protein; ESR, erthrocyte sedimentation rate; ALT, alanine aminotransferase; AST, aspartate aminotransferase; IFN, interferon; IL, interleukin; Ruxo, ruxolitinib; sJIA, systemic juvenile idiopathic arthritis; SLE, systemic lupus erythematosus; CTDs, connective tissue diseases.

Bold values indicate statistical significance.

**Table 2:** Clinical characteristics and outcomes patients with RD-MAS.

Patient	Underlying disease	Disease onset (years)	MAS diagnosis (years)	White blood cells (10^9/L)	Neutrophils (10^9/L)	Hemoglobin (g/L)	Platelets (10^9/L)	CRP (mg/L)	ESR (mm/h)	Ferritin (ng/mL)	Triglycerides (mmol/L)	Fibrinogen (g/L)	ALT (IU/L)	AST (IU/L)	Concomitant treatments at initiation	Initial treatment of GCs (equivalent dose of Pred, mg/kg/day)	ruxolitinib dosing	Initiation of ruxolitinib (days)	ruxolitinib duration (years)	Response at last follow-up	Treatments at last follow-up	Dose of GCs at last follow up (equivalent dose of Pred, mg/kg/day)
1	sJIA	7.92	7.92	14.11	12.26	122	191	172.6	27	22614.9	1.32	7.58	42	49	CAN	6	2.5mg bid→ 5mg bid	17	0.62	CR	None	0
2	sJIA	8.83	8.83	34.28	31.92	110	537	136.8	79	9304	0.95	7.77	6	22	TCZ	2	5mg bid	7	0.9	CR	TCZ	0
3	JDM	8.92	9.08	5.67	3.97	116	238	0.2	6	1453	0.82	1.77	389	275	CTX	3	5mg bid→15mg/day	15	0.23	CR	TOF+MTX	0
4	sJIA	10.17	10.17	12.83	11.14	92	206	92.8	51	11054	0.92	4.67	13	41	TCZ+MTX	20	7.5mg bid	12	0.92	CR	TCZ+MTX	0
5	sJIA	9.67	10.08	5.06	4.26	126	164	82.8	7	8762	1.18	2.56	118	323	TCZ→ CAN	20	10mg bid	26	0.04	CR	CAN	0
6	sJIA	13.17	13.33	16.33	14.68	125	447	35.8	80	2973	1.3	3.53	49	31	TCZ	1	5mg bid	5	0.23	CR	TCZ+MTX	0
7	Vasculitis	0.25	0.25	17.36	15.41	76	79	191.2	21	1447	0.8	4.69	15	12	CTX	10	2.5mg qd	17	0.58	CR	AZA	0
8	sJIA	2.92	2.92	38.46	33.46	100	797	159.7	87	5252.85	0.83	7.84	14	26	CAN+MTX	2	5mg bid	6	0.46	CR	None	0
9	SLE	9.83	9.83	1.64	0.61	71	168	0.2	27	983	3.14	3.68	32	64	MMF	5	5mg qd	5	0.54	CR	BEL+MMF	0
10	sJIA	3.33	3.33	16.32	14.09	106	344	105.8	92	2299.42	1.3	9.26	40	74	TCZ→ CAN	2	5mg bid	25	0.23	CR	CAN	0
11	SLE	14.25	14.33	1.62	0.81	117	55	0.3	3	1920	1.77	1.43	347	600	BEL+MMF	2	None	None	None	CR	BEL+MMF	0.2
12	SS	11.63	11.67	2.99	1.46	109	189	7.2	25	783	1.23	3.69	48	57	MMF	0.7	None	None	None	CR	TAC	0
13	sJIA	9.17	9.17	19.91	16.37	101	474	76.3	48	3890	1.91	5.6	16	54	CAN+MTX	2	None	None	None	CR	None	0
14	SS	4.79	4.83	2.96	1.38	94	268	14.67	73	33315.6	2.91	2.17	2193	3568	CsA	2	None	None	None	CR	MMF	0.2
15	SLE	10	10.08	1.84	0.71	75	42	0.2	3	695	2.79	1.19	136	588	CTX	3	None	None	None	CR	BEL+MMF	0.2
16	sJIA	6.17	6.25	13.85	13	102	277	80	49	11033	1.28	4.17	34	89	TCZ	4	None	None	None	CR	TCZ	0
17	sJIA	2.17	2.25	18.06	13.52	83	518	59.34	44	3571.11	1.37	4.94	7	44	TCZ+MTX	2	None	None	None	CR	TCZ+MTX	0.4
18	SS	4.25	4.42	2.58	1.76	114	150	18.37	10	2158.56	0.96	2.4	76	154	CTX	2	None	None	None	PR	MTX	0.2
19	SLE	12.71	12.75	1.41	0.72	117	83	0.7	33	7936.95	2.55	1.67	390.5	1707	MMF	1	None	None	None	CR	MMF	0.06
20	SLE	10.33	10.33	2.19	1.26	113	123	5.75	10	936.01	1.59	2.93	71	75	BEL+MMF	2	None	None	None	CR	MMF	0.1
21	SLE	5.5	5.67	2.69	1.13	98	160	0.36	37	1095.62	0.83	1.99	244	825	BEL+MMF	3	None	None	None	CR	BEL+MMF	0.2

sJIA, systemic juvenile idiopathic arthritis; JDM, juvenile dermatomyositis; SLE, systemic lupus erythematosus; SS, Sjögren’s syndrome; CRP, C-reactive protein; ESR, erthrocyte sedimentation rate; ALT, alanine aminotransferase; AST, aspartate aminotransferase; CAN, canakinumab; TCZ, Tocilizumab; MTX, methotrexate; CTX, cyclophosphamide; TOF, tofacitinib; AZA, azathioprine; MMF, mycophenolate mofetil; BEL, belimumab; TAC, tacrolimus; CsA, cyclosporine; GC, glucocorticoids; Pred, prednisone; bid, twice daily; qd, once daily; CR, complete response; PR, partial response.

In the ruxolitinib group, 10 patients (6 males, 4 females) had underlying diagnoses of systemic juvenile idiopathic arthritis (sJIA, n = 7), systemic lupus erythematosus (SLE, n = 1), or other connective tissue diseases (n = 2), with a median age at RD-MAS onset of 8.9 years (IQR, 3.2-9.9). All patients presented with fever at diagnosis. Bone marrow hemophagocytosis was observed in 20% (2/10) of patients, and 30% (3/10) exhibited cytopenia affecting two or three lineages. Serum ferritin levels were markedly elevated in all patients, with a median level of 4112.9 ng/mL (IQR, 1451.5-9741.5). Elevated levels of ALT, aspartate aminotransferase (AST), and lactate dehydrogenase (LDH) were observed in the majority of cases. Concomitant therapies in this group included canakinumab (CAN) (n = 4, 40%), tocilizumab (TCZ) (n = 5, 50%), methotrexate (MTX) (n=3, 30%), cyclophosphamide (CTX) (n=3, 30%), and mycophenolate mofetil (MMF) (n = 1, 10%) (**Table 2**). Intravenous immunoglobulin (IVIG) was administered to 3 patients (n=3, 30%).

In the non-ruxolitinib group, 11 patients (3 males, 8 females) had underlying diagnoses of sJIA (n=3), SLE (n=5) or other CTDs (n=3). The two cohorts exhibited broadly similar major clinical features of MAS, including fever, cytopenia, hyperferritinemia, and elevated liver enzymes. However, patients in the ruxolitinib group had significantly higher white blood cell and neutrophil counts, as well as lower AST levels (**Table 1**). Although SLE and other connective tissue diseases (CTDs) were more prevalent in the non-ruxolitinib group (*p* = 0.149 and *p* = 1, respectively), and sJIA was more common in the ruxolitinib group (*p* = 0.086), none of these between-group differences reached statistical significance. In this control group, patients were managed with glucocorticoids combined with biologic or conventional disease-modifying antirheumatic drug (DMARDs), including TCZ (n=3, 27.3%), CAN (n=1, 9.1%), MTX (n=2, 18.2%), MMF (n=5, 45.5%) and belimumab (BEL) (n=3, 27.3%). Treatment regimens were individualized according to the underlying rheumatic disease and physician discretion (**Table 2**).

### Treatment response and safety

**Figure 2:**
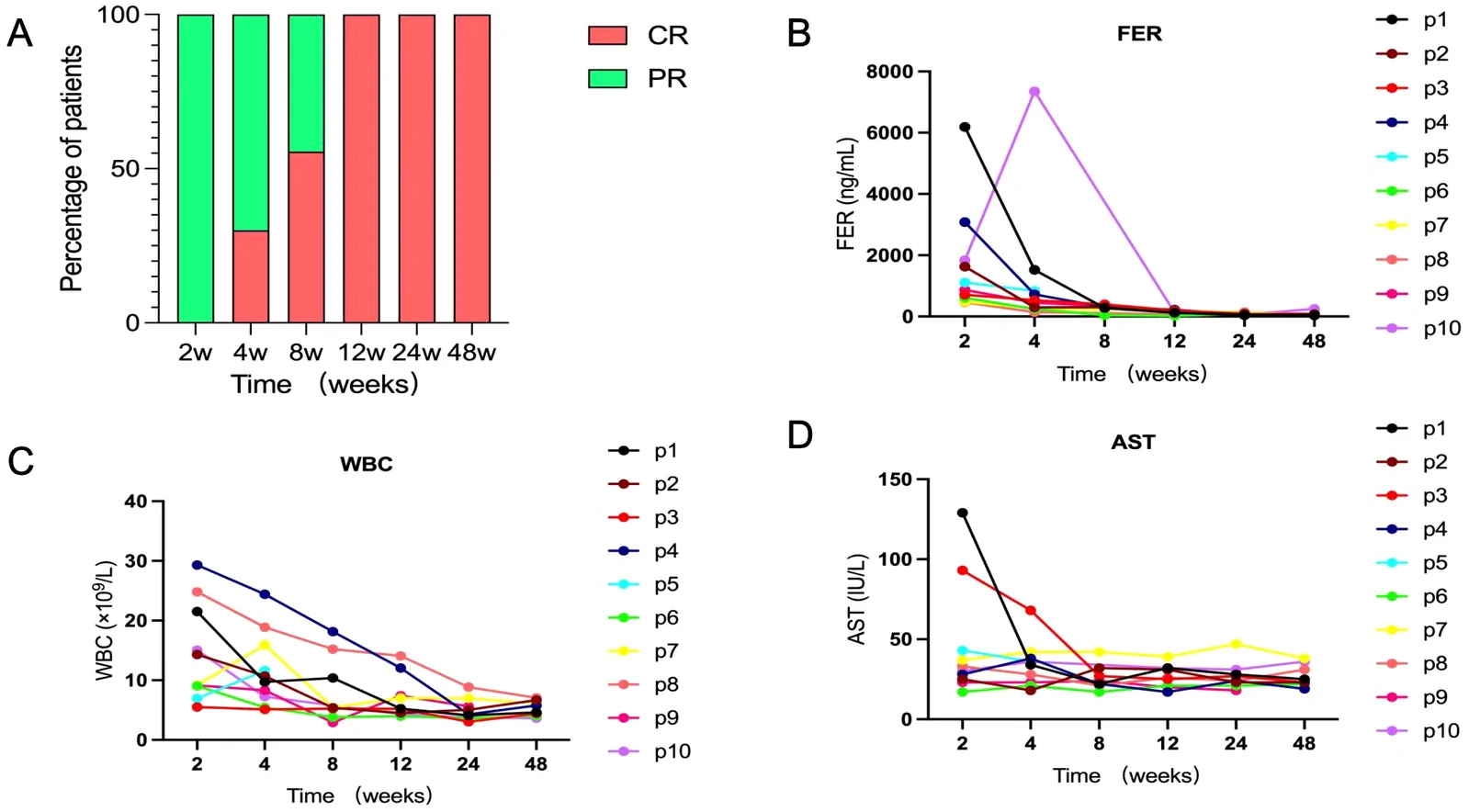
Outcomes of ruxolitinib therapy. **(A)** Percentage of response in patients with RD-MAS after ruxolitinib treatment. **(B–D)** Temporal changes in laboratory measures after ruxolitinib treatment. The time axis in **(B–D)** is not linearly scaled. CR, complete remission; PR, partial remission; WBC, white blood cells; FER, ferritin; AST, alanine aminotransferase.

The therapeutic response to ruxolitinib is shown in **Figure 2A**. The median interval between RD-MAS diagnosis and ruxolitinib initiation was 13.5 (6–17) days. Patients in the ruxolitinib group received oral ruxolitinib at a median dose of 10 mg/day (IQR, 6.25-13.75), with a median treatment duration of 0.5 years (IQR, 0.2-0.6). Dose escalation was required in 20% (2/10) of patients due to persistent hyperferritinemia (**Table 2**).

Patients treated with ruxolitinib demonstrated progressive clinical improvement over time, with an increasing proportion achieving CR during follow-up. By week 4, 30% (3/10) of patients in the ruxolitinib group achieved CR, and all patients achieved CR by week 48. PR in 70% (7/10) was observed during the early treatment phase, followed by gradual conversion to CR. By week 24, 20% (2/10) of patients discontinued ruxolitinib after achieving CR, and no relapse was observed during the follow-up period. By week 48, all patients had discontinued ruxolitinib and remained in sustained remission. Dynamic changes in laboratory parameters following ruxolitinib treatment are presented in **Figures 2B–D**. Key inflammatory markers, including WBC, FER, and AST, showed progressive normalization after treatments.

**Figure 3:**
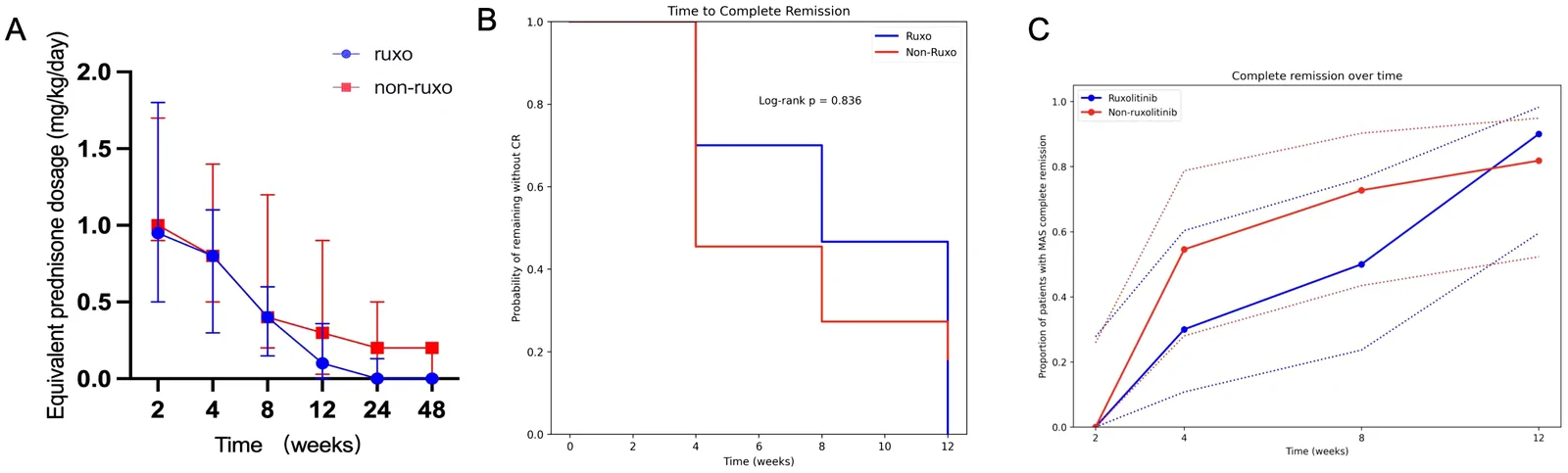
Glucocorticoid tapering and time to complete remission in two groups. **(A)** Median prednisone-equivalent dosage (mg/kg/day) over time after treatments. **(B)** Time to complete remission in pediatric patients with refractory disease-associated macrophage activation syndrome (RD-MAS). **(C)** Proportion of patients with RD-MAS achieving complete remission over time after treatments. The time axis in panel **(A)** is not linearly scaled. ruxo, ruxolitinib group; non-ruxo, non-ruxolitinib group; RD-MAS, refractory disease-associated macrophage activation syndrome.

Kaplan–Meier analysis of time to CR is shown in **Figure 3B**. Both groups demonstrated increasing remission over time. However, no statistically significant difference in time to CR was observed between two groups (log-rank *p* = 0.836). The proportion of patients achieving CR over time is shown in **Figure 3C**. The non-ruxolitinib group showed a numerically higher CR rate during the early follow-up period, whereas the ruxolitinib group demonstrated a gradual increase and reached a comparable remission rate by week 12.

Adverse events (AEs) were minimal and predominantly mild. The most common AEs in the ruxolitinib group was mild infection, particularly respiratory tract infection. Mild infections occurred in 5/10 (50.0%) patients in the ruxolitinib group and 6/11 (54.5%) patients in the non-ruxolitinib group, this difference was not statistically significant (*p* = 0.527). No unexpected adverse events, including serious infections or thrombotic events, were reported.

### Glucocorticoid-sparing effect

Changes in glucocorticoid dosage over time are shown in **Figure 3A**. All patients received high-dose glucocorticoids at the initiation of ruxolinitib therapy (**Tables 1**, **2**), and no significant difference was observed between the two groups (p =0.062). In the ruxolitinib group, the median prednisone-equivalent dose decreased markedly during follow-up, dropping from an initial dose of 4 mg/kg/day to 0.4 mg/kg/day by week 8, representing a substantial reduction in glucocorticoid exposure. Importantly, this glucocorticoid-sparing effect was continuously sustained throughout the follow-up period. By week 48, all patients in the ruxolitinib group had successfully discontinued glucocorticoids while maintaining CR. In contrast, 72.7% (8/11) of patients in the non-ruxolitinib group still required low-dose glucocorticoid therapy at week 48, with a median dose of 0.2 mg/kg/day (IQR 0.03-0.20). The ruxolitinib group exhibited a significantly lower median cumulative steroid dose (130.56 mg/kg, IQR: 112.32–158.70) compared with the non-ruxolitinib group (196.84 mg/kg, IQR: 165.21–228.36, *p* < 0.05) over 48 weeks of follow-up. A statistically significant difference in dynamic glucocorticoid dosage was consistently observed during follow-up (*p* = 0.021), which became more evident at later time points.

## Discussion

In this retrospective study, we evaluated the efficacy, safety, and glucocorticoid-sparing effects of ruxolitinib in pediatric patients with RD-MAS in a real-world setting. The principal observation of this study was that patients treated with ruxolitinib appeared to require lower cumulative glucocorticoid exposure while maintaining disease control. Although ruxolitinib did not significantly shorten the time to complete remission compared with conventional treatment, patients receiving ruxolitinib achieved progressive clinical and laboratory improvement and were able to discontinue glucocorticoids more successfully during follow-up.

The most clinically relevant finding of our study was the marked glucocorticoid-sparing effect associated with ruxolitinib treatment. Glucocorticoid doses declined rapidly after ruxolitinib initiation and continued to decrease throughout follow-up, with all patients successfully discontinuing glucocorticoids by week 48 while maintaining remission. In contrast, most patients in the non-ruxolitinib group remained on low-dose glucocorticoids at the final follow-up. Given the well-recognized adverse consequences of prolonged glucocorticoid exposure in children, including growth impairment, metabolic disturbances, and increased infection risk (10–12), these findings highlight the potential value of ruxolitinib as a steroid-sparing therapeutic strategy in pediatric RD-MAS.

Our findings are consistent with accumulating evidence supporting JAK inhibition in hyperinflammatory syndromes. Previous studies have demonstrated the efficacy of ruxolitinib in both primary HLH and MAS (13–18). Notably, Zhang et al. reported a prospective open-label pilot study in which ruxolitinib was used as first-line therapy in 12 children with secondary HLH, resulting in rapid fever resolution, significant improvement in inflammatory markers, and favorable tolerability (18). Similar to their findings, patients in our cohort showed sustained clinical and laboratory improvement without major safety concerns. However, unlike the Zhang study, which evaluated front-line treatment in secondary HLH, including a substantial proportion of EBV-HLH cases, our cohort specifically focused on RD-MAS and included a comparative non-ruxolitinib group, offering additional insight into the potential role of ruxolitinib in this distinct clinical setting.

The optimal dosing strategy for pediatric RD-MAS remains uncertain. In our cohort, the median daily dose of ruxolitinib was 10 mg, and dose escalation was required in two patients because of persistent hyperferritinemia, suggesting that individualized dose adjustment may be necessary in patients with ongoing inflammation activity. Adverse events in our cohort were minimal and predominantly mild. mild respiratory tract infection was the most common adverse event, and no additional safety concerns were observed in patients requiring dose escalation. Although long-term pediatric safety data remain limited, several adverse events of special interest described in adults have not been observed to date in children. Likewise, recent pharmacovigilance analyses have not identified any specific organ-related disproportionality signals among pregnant women exposed to JAK inhibitors (19). Further prospective studies are needed to define the most effective dosing regimen, treatment duration and long-term safety for pediatric RD-MAS.

We did not observe a statistically significant difference in time to CR between the ruxolitinib and non-ruxolitinib groups. This finding may be explained by the limited sample size, the retrospective study design, and baseline heterogeneity between treatment groups. In addition, several patients received concomitant conventional or biologic DMARDs for their underlying rheumatic disease, which may have contributed to disease control independently of ruxolitinib. Nevertheless, the sustained reduction in glucocorticoid exposure observed in the ruxolitinib group suggests a clinically meaningful steroid-sparing effect beyond remission induction.

Ruxolitinib exhibits substantial interindividual pharmacokinetic variability: a recent population pharmacokinetic studies demonstrated exposure–toxicity relationships in routine clinical practice, with higher drug exposure associated with an increased risk of adverse events (20). Clinically relevant drug–drug interactions should also be considered, particularly with concomitant use of strong CYP3A4 inhibitors, including macrolide antibiotics, azole antifungals, or pharmacokinetic enhancers (e.g., ritonavir) (21), which can significantly increase ruxolitinib exposure and the risk of concentration-dependent toxicity. Recent advances in LC–MS/MS analytical methods now enable accurate plasma quantification of ruxolitinib (22), creating opportunities for therapeutic drug monitoring and model-informed precision dosing, including physiologically based pharmacokinetic (PBPK) approaches. Although pharmacokinetic monitoring was not performed in the present cohort, systematic assessment of concomitant medications together with prospective pharmacokinetic studies may help optimize individualized dosing and improve the safety of ruxolitinib therapy in pediatric patients.

Several limitations should be acknowledged. This was a single-center retrospective study with a small sample size and inherent risks of selection bias and residual confounding. Treatment allocation was based on physician discretion, drug availability, and economic considerations rather than randomization, and heterogeneity in underlying diseases as well as concomitant therapies may have confounded the outcomes. In addition, several baseline laboratory variables differed between groups, which may have influenced treatment response and contributed to residual confounding. Furthermore, treatment response was assessed retrospectively. Due to the exploratory nature of the study, our findings require cautious interpretation. Therefore, prospective multicenter studies are required to validate our findings and better define the role of ruxolitinib in pediatric RD-MAS.

## Conclusion

Ruxolitinib-based therapy was associated with sustained disease control and a significant glucocorticoid-sparing effect in pediatric RD-MAS. Although no superiority in time to complete remission was observed compared with conventional therapy, ruxolitinib enabled successful glucocorticoid discontinuation in all treated patients without increased infectious complications. These findings suggest that ruxolitinib may serve as a promising steroid-sparing strategy with favorable efficacy and safety for pediatric RD-MAS, although larger prospective studies are warranted to confirm these results.

## Data Availability

The original contributions presented in the study are included in the article/supplementary material. Further inquiries can be directed to the corresponding author.
